# Demography, common disorders, cause-specific mortality and life expectancy of Ragdoll cats under primary veterinary care during 2019 in the UK

**DOI:** 10.1186/s40575-025-00148-9

**Published:** 2025-11-05

**Authors:** Dan G. O’Neill, Olivia Bass, Dave C. Brodbelt, David B. Church, Karolina S. Engdahl

**Affiliations:** 1 Pathobiology and Population Sciences, The Royal Veterinary College, Hawkshead Lane, North Mymms, Hatfield, AL9 7TA Herts UK; 2 Clinical Sciences and Services, The Royal Veterinary College, Hawkshead Lane, North Mymms, Hatfield, AL9 7TA Herts UK; 3 Department of Clinical Sciences, Swedish University of Agricultural Sciences, PO Box 7054, Uppsala, 750 07 Sweden

**Keywords:** VetCompass, Ragdoll, Electronic health record, Breed, Cat, Pedigree

## Abstract

**Objectives:**

This study aimed to characterise the demography, common disorders, cause-specific mortality and life expectancy of the general population of Ragdoll cats under primary veterinary care in the UK in 2019.

**Methods:**

The study used a retrospective cohort design of all cats under UK primary veterinary care within VetCompass Programme during 2019. The clinical records of a random sample of 2,025 Ragdolls were reviewed to identify every disorder recorded during 2019.

**Results:**

Ragdolls comprised 21,358 (1.70%) of 1,254,484 study cats. Annual proportional birth rates showed increasing Ragdoll popularity from 0.70% of all cats born in 2005 to 3.72% in 2019. Median adult Ragdoll bodyweight was 4.46 kg (IQR 3.79–5.19). The most prevalent precise-level precision disorders recorded were periodontal disease (8.84%, 95% CI: 7.60-10.08), diarrhoea (7.11%, 95% CI: 5.99–8.23) and obesity (6.91%, 95% CI: 5.81–8.02). The most common disorder groups were dental (14.52%, 95% CI: 12.98–16.05), enteropathy (13.48%, 95% CI: 11.99–14.97), skin (7.75%, 95% CI: 6.59–8.92) and obesity (6.91%, 95% CI: 5.81–8.02). From 133 Ragdolls that died during the study period, the median age at death was 12.85 years (IQR 5.71–16.11). The most common causes of death at group level precision were kidney disorder (21.18%, 95% CI: 12.49–29.86) and poor quality of life (12.94%, 95% CI: 5.81–20.08).

**Supplementary Information:**

The online version contains supplementary material available at 10.1186/s40575-025-00148-9.

## Background

The Ragdoll is a large, semi-longhaired cat breed invented in the US in the 1960s reportedly by linebreeding from a pure white longhaired cat of Angora appearance, a seal mitted male and a solid black cat. The breed was first introduced into the UK in 1981 [1]. The Ragdoll name originates from their typically placid, friendly nature and tendency to go limp ‘like a ragdoll’ when picked up [2]. The Ragdoll is recognised as a pedigree breed by the UK The Governing Council of the Cat Fancy (GCCF) but there is limited evidence on the popularity and health of the wider pet population of Ragdoll cats in the UK.

In the UK, the Ragdoll was the second most common pure cat breed under primary veterinary care in 2019, accounting for 1.8% of all the cats, and with this proportion having doubled from the 0.9% reported for 2014 [3, 4]. The Cats Protection charity reported the Ragdoll as the equal-second most commonly acquired cat breed in 2023 in the UK, accounting for 4% of all cats acquired that year [5]. In Australia, the Ragdoll was the second most common cat breed under primary veterinary care from 2005 to 2015, accounting for 5.1% of all cats [6]. However, for dogs, rising public demand for specific pure breeds is recognised to often lead to new and serious health and welfare challenges for these animals. These issues have been evidenced in rapidly popularised dog breeds such as French Bulldogs where effects from high levels of inbreeding and extreme conformation compounded by direct suffering from farming these animals on industrial scales have created a brachycephalic health crisis that is proving very challenging to resolve [7, 8]. In anticipation of similar potential welfare issues for rapidly popularised cat breeds, improved data on Ragdoll proportional ownership among the wider population of cats and the demography of these cats, e.g. bodyweight, could contribute to better understanding and management of changing welfare challenges over time.

Despite high ownership, there is limited published evidence on overall disorder prevalence and predisposition for the Ragdoll. The UK GCCF lists only two health issues for the Ragdoll: hypertrophic cardiomyopathy and blood group incompatibility between Ragdoll parents [1]. However, a disorder predisposition textbook that reviewed the overall scientific literature identified published evidence for 13 disorders in Ragdolls [9]. In contrast, analysis of pet insurance data from 1999 to 2006 in Sweden ranked the Ragdoll with only the 13th highest incidence rate for having at least one veterinary care event from 20 cat breeds assessed [10]. However, those authors did suggest that their results were biased toward apparently better health because the Ragdoll had recently increased in popularity and therefore could be younger, on average, than breeds with a more stable popularity such as the Siamese. Overall, it appears that much of the current evidence on disorder risk in Ragdolls is limited by small sample size and selection bias in the study populations, and with many of these published studies now being quite dated.

Reliable information about mortality and life expectancy can contribute to improved understanding of the health and welfare within individual cat breeds and can assist to identify changes in the general health of the population over time [11]. A mortality study of cats under UK primary veterinary care between 2009 and 2012 identified median Ragdoll age at death of 10.1 years compared to 14.0 years cats overall, suggesting some evidence of poor overall breed health [12]. Analysis of pet insurance data from 2003 to 2006 in Sweden identified the Ragdoll with the lowest probability of survival to 10 years from the 9 common cat breeds assessed [13]. However, these short life expectancy results could be biased downwards because the population of Ragdolls studied was more youthful overall than for other breeds and therefore offered a higher proportion of younger Ragdoll cats to contribute death data in the first place.

Using veterinary clinical data from the VetCompass Programme [14], this study aimed to characterise the demography, common disorders, cause-specific mortality and life expectancy of the general population of Ragdoll cats under primary veterinary care in the UK in 2019. Specific focus was placed on disorder risk associations with sex and age. These results could provide an evidence base to assist reforms in breeding and purchasing practices for Ragdoll cats and ultimately contribute to improved health and welfare.

## Materials and methods

The study population included all cats under primary veterinary care at clinics participating in the VetCompass Programme during 2019. Cats under veterinary care were defined as those with at least one electronic health record [EHR] (free-text clinical note, treatment, or bodyweight) recorded during 2019 from their entire available clinical records at any date. VetCompass data fields available for the current study from the entire available clinical records at any date included fixed value variables of unique animal identifier, species, breed, date of birth, sex and neuter status along with time-varying variables of bodyweight, free-form text clinical notes and treatment with relevant dates.

Cats recorded as Ragdoll breed were categorised as Ragdolls while all remaining cats were categorised as non-Ragdolls. *All-age Bodyweight* (kg) described all available bodyweight and date combinations. *Adult Bodyweight* (kg) described the mean bodyweight recorded from all bodyweight data for cats aged over 9 months and was categorised into 4 groups (< 3, 3 to < 4, 4 to < 5, ≥ 5). *Neuter* described the status of the cat (entire or neutered) recorded at the final EHR. *Age* (years) described the age at the final date under veterinary care during 2019 (December 31 st, 2019) and was categorised into 6 groups (< 2, 2 to < 4, 4 to < 6, 6 to < 8, 8 to < 10, and ≥ 10.0).

A retrospective cohort study design was used to estimate the one-year period prevalence of the most diagnosed disorders of Ragdoll cats from a population of 1,254,484 cats across all breeds under primary veterinary care during 2019 at practices participating within the VetCompass Programme. Sample size calculations estimated that 1,822 cats were needed to report a disorder with 5.0% expected prevalence with 95% confidence level for a 1.0% margin of error from an estimated UK population of 11 million cats [15, 16]. Ethical approval was given by the Royal Veterinary College Social Science Research Ethical Review Board (SSRERB) (reference number SR2018-1652).

The VetCompass study design, data extraction and analysis methods followed those previously published [3]. The EHRs of a random sample from all available Ragdolls were manually reviewed in detail. Each disorder event was followed over time in the cohort data source to identify the most definitive diagnosis term recorded for every disorder recorded as existing during 2019 and to link this to the most appropriate VeNom term as previously described [17]. The extracted diagnosis terms were mapped to a dual hierarchy of precision for analysis: precise-level precision and grouped-level precision [17]. The EHRs were accessed via the VetCompass Programme online user interface (VetCompass.org). Precise-level diagnostic terms described the original extracted terms at the maximal diagnostic precision recorded within the clinical notes (e.g. *inflammatory bowel disease* remained as *inflammatory bowel disease*). Grouped-level precision terms mapped the original diagnosis terms to a general level of diagnostic precision (e.g. *inflammatory bowel disease* mapped to *enteropathy)*. Disorders described within the clinical notes using presenting sign terms (e.g. ‘vomiting’ or ‘vomiting and diarrhoea’) without a formal clinical diagnostic term were included using the first sign listed (e.g. vomiting). Elective (e.g. neutering) or prophylactic (e.g. vaccination) clinical events were excluded. No distinction was made between pre-existing and incident disorder presentations. Cause-specific mortality data (recorded cause, date and method of death) were extracted on all deaths at any date during the available EHRs.

### Statistical analysis

Following data checking for internal validity and cleaning in Excel (Microsoft Office Excel 2013, Microsoft Corp.), analyses were conducted using R version 4.2.1 [18]. Annual proportional birth rates described the relative proportion of Ragdolls compared with all cats that were born in each year from 2005 to 2019 from the cohort under veterinary care in 2019. The figure illustrating annual proportional birth rates was generated with the R package ggplot2 [19]. All bodyweight data with their associated dates at any age of cat were used to generate individual bodyweight growth curves for male and female Ragdolls by plotting age-specific bodyweights overlaid with a cross medians line using the R package ggplot2 [19].

One-year period prevalence values were reported along with 95% confidence intervals (CI) that described the probability of diagnosis at least once during 2019. The CI estimates were derived from standard errors based on approximation to the normal distribution (Wald CI) for disorders with ten or more events [20] or the Wilson approximation method for disorders with fewer than ten events [21], using the binom.approx() and binom.wilson() functions from the R package epitools [22]. Prevalence values were reported overall and separately for males and females. Median age (years) as defined above was reported for each of the most common diagnoses at precise-level and group-level. Prevalence values for the top 20 most common group-level disorders were reported in three age groups (< 2 years, 2–6 years, and ≥ 7 years), and the disorders were ranked within each age group based on the prevalence values. Prevalence values were compared between age groups using the chi-square test. The lists of the 10 most common group-level disorders in each age group were merged to generate one master list of 15 disorders. The annual prevalence of each these 15 disorders is presented using loess curves in a figure generated with the R packages ggplot2, cowplot, and ggpubr [19, 23, 24]. Cats aged 16 or older were grouped together in the plot.

A combination of the Shapiro-Wilk test and visual assessment of histograms was used to assess normality of continuous variables. The two-proportion z-test was used to compare proportions, chi-square test to compare categorical variables, and the Mann-Whitney U test to compare continuous variables as these deviated from normality [20]. Statistical significance was set at the 5% level. The current statistical analyses were univariable so potential impacts from confounding should be considered when interpreting the results [25]. The current analysis also reported on many separate statistical tests without applying a correction factor to adjust for multiple testing [26]. Therefore the possibility of false positives should be acknowledged when interpreting individual test results and readers are encouraged to consider each individual result as hypothesis-generating rather than hypothesis-confirmatory [27].

## Results

### Demography

**Fig. 1:**
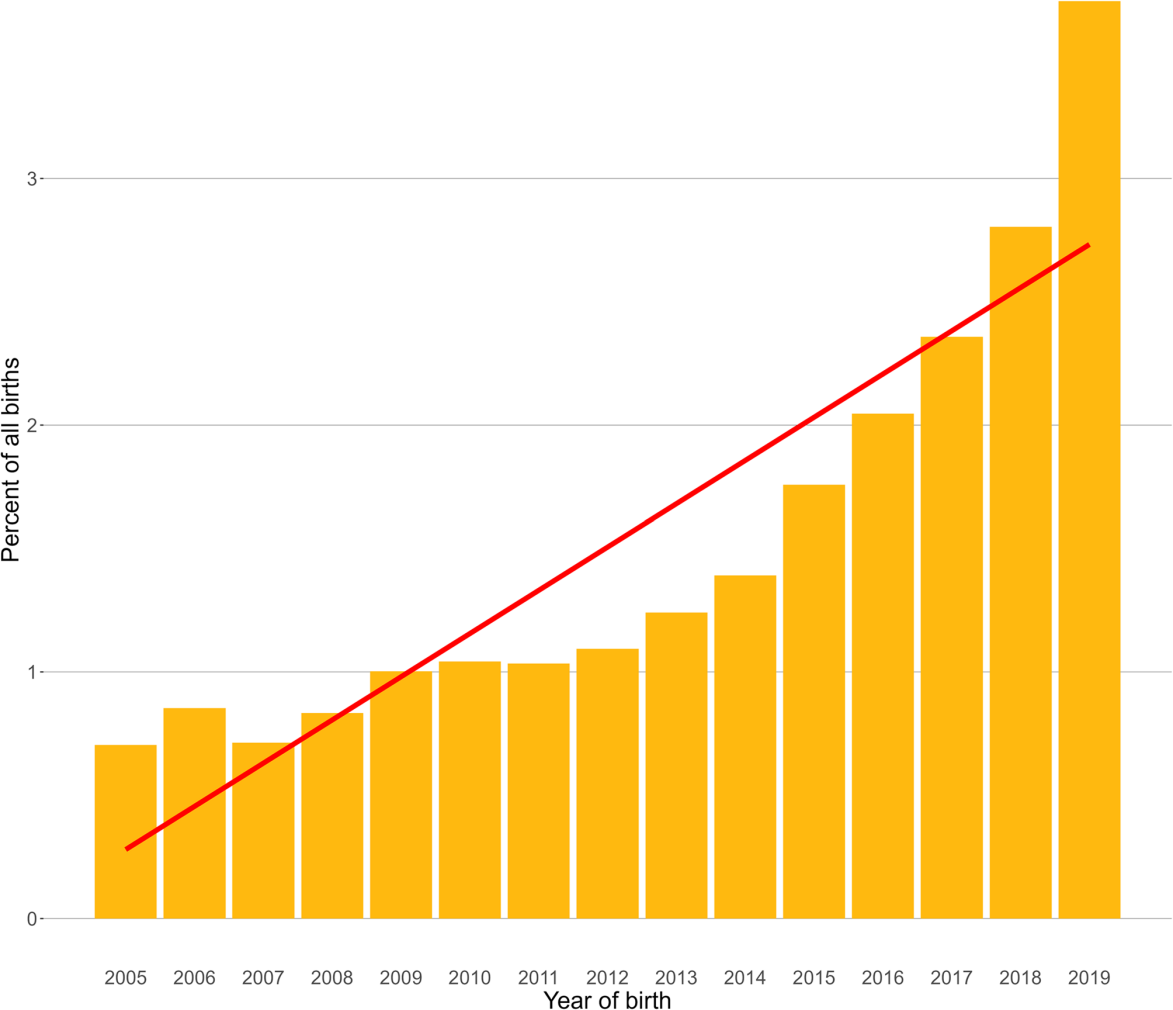
Annual proportional birth rates (2005–2019) with linear trendline for Ragdolls (*n =* 21,358) among all cats (*n =* 1,254,484) under UK primary veterinary care from January 1st 2019 to December 31st, 2019 at practices participating in the VetCompass Programme

The study population of 1,254,484 cats under veterinary care during 2019 in the UK included 21,358 (1.70%) Ragdolls. Of the Ragdolls with information available, 9,691 (45.37%) were females and 9,667 (45.26%) were neutered (Table 1). Males were statistically significantly more likely than females to be recorded as neutered (41.77% female and 49.02% male, respectively, chi-square test: *P* < 0.001). The overall median age was 2.63 years (interquartile range (IQR) 1.20–6.01, range 0.06–23.18). Annual proportional birth rates showed an increasing breed popularity during 2005–2019, from 0.70% of all cats born in 2005 to 3.72% in 2019 (Fig. 1).

**Table 1:** Demography of Ragdoll cats under primary veterinary care at practices participating in the VetCompass programme in the UK from January 1st to December 31st, 2019. *Counts cover cats with available data

Variable	Category	Female (%)*	Male (%)*	Total (%)*
Neuter		4,048 (41.77)	5,619 (49.02)	9,667 (45.26)
Adult bodyweight (kg)	< 3	600 (8.16)	55 (0.62)	661 (4.07)
	3 to < 4	3,696 (50.26)	896 (10.14)	4,615 (28.41)
	4 to < 5	2,419 (32.89)	3,576 (40.47)	6,008 (36.98)
	> 5	639 (8.69)	4,310 (48.77)	4,963 (30.55)
Age (years)	< 2	3,953 (41.18)	4,730 (41.50)	8,822 (41.64)
	2 to < 4	2,061 (21.47)	2,400 (21.06)	4,477 (21.13)
	4 to < 6	1,190 (12.40)	1,390 (12.20)	2,589 (12.22)
	6 to < 8	731 (7.62)	918 (8.05)	1,652 (7.80)
	8 to < 10	573 (5.97)	723 (6.34)	1,310 (6.18)
	≥ 10	1,091 (11.37)	1,237 (10.85)	2,338 (11.03)

**Fig. 2:**
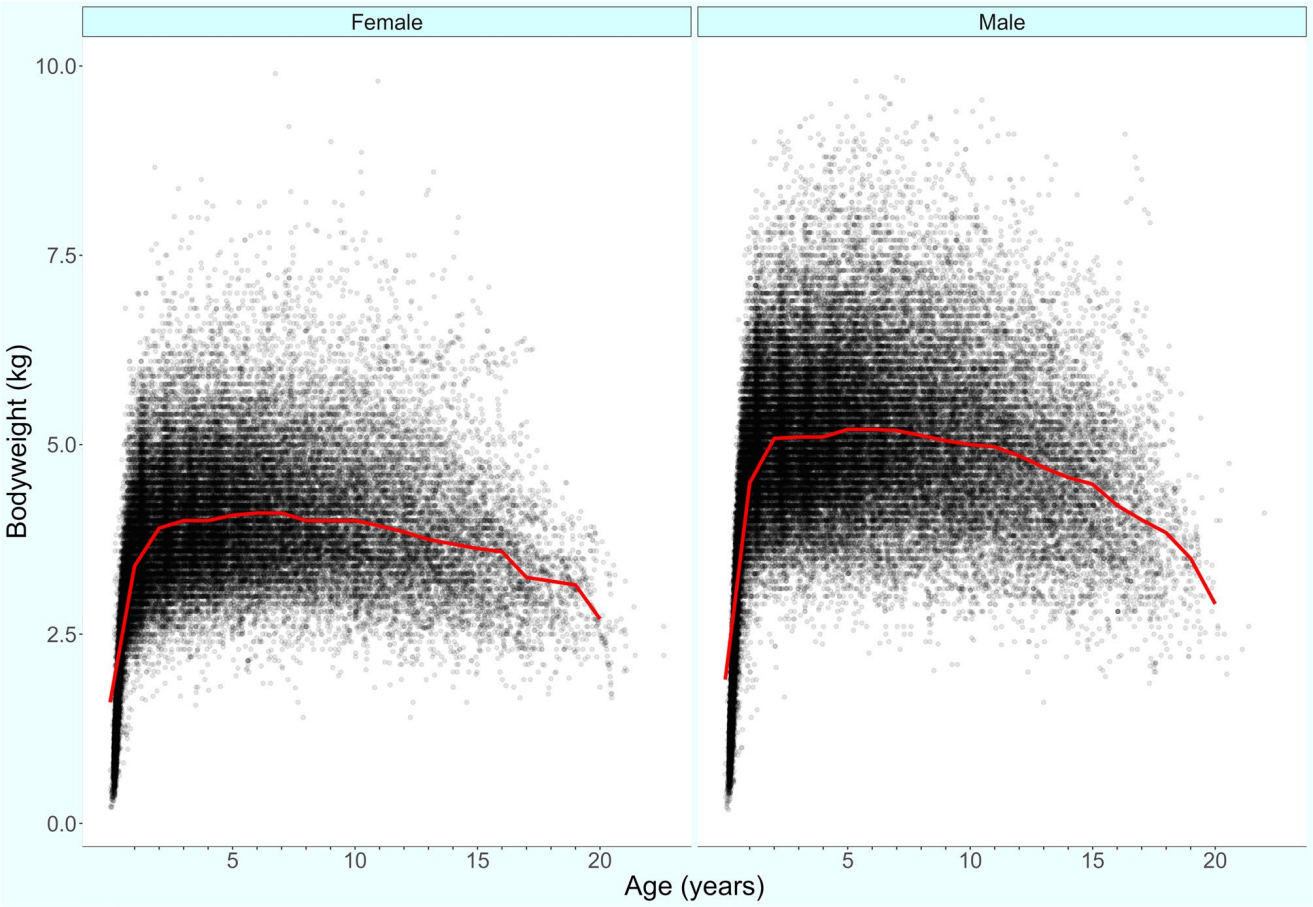
Bodyweight at different life stages with a cross medians line plot for female (*n =* 8,586) and male (*n =* 10,390) Ragdoll cats under UK primary veterinary care from January 1 st 2019 to December 31 st, 2019 at practices participating in the VetCompass Programme

The median adult bodyweight was 4.46 kg (IQR 3.79–5.19, range 1.46–9.80). Males (4.97 kg, IQR 4.42–5.60, range 1.60–9.51) were statistically significantly heavier than females (3.83 kg, IQR 3.40–4.38, range 1.46–9.80) (Mann-Whitney U test: *P* < 0.001). The median bodyweight across all ages was statistically significantly higher in males (4.34, IQR 3.48–5.10, range 0.20–9.51) than in females (3.39, IQR 2.76–3.99, range 0.22–9.80) (Mann-Whitney U test: *P* < 0.001). Bodyweight curves based on 70,518 bodyweight values in 10,390 males and 54,837 bodyweight values in 8,586 females showed that the Ragdolls grow rapidly during their first year and continue to gain weight until around two years of age (Fig. 2).

The proportional completeness for each variable was sex 99.04%, neuter 99.04%, mean adult bodyweight 76.07%, and age 99.20%.

### Disorder prevalence

The EHRs from a random sample of 2,025 of the 21,358 available Ragdolls (9.48%) were manually reviewed to extract information on all disorders recorded during 2019. Of these Ragdolls, 1,241 (61.28%) had at least one disorder recorded during 2019, while the others received only prophylactic care or no active veterinary care during 2019. During 2019, there were 2,203 unique disorder events reported. The median annual disorder count was 1 (IQR 0–2, range 0–9) disorder per Ragdoll. The annual disorder count was statistically significantly higher in males (median count 1, IQR 0–2, range 0–8) than females (median count 1, IQR 0–2, range 0–9) (Mann-Whitney U test, *P* = 0.047).

There were 277 distinct precise-level disorder terms reported during 2019. The most prevalent precise-level precision disorders recorded were periodontal disease (*n* = 179, prevalence 8.84%, 95% CI: 7.60–10.08), diarrhoea (*n* = 144, prevalence 7.11%, 95% CI: 5.99–8.23), obesity (*n* = 140, prevalence 6.91%, 95% CI: 5.81–8.02), overgrown nail(s) (*n* = 115, prevalence 5.68%, 95% CI: 4.67–6.69), and dental disease (*n* = 113, prevalence 5.58%, 95% CI: 4.58–6.58) (Table 2). Among the 32 most common precise-level disorders, females had statistically significantly higher probability of postoperative wound complications and aural discharge, while males had statistically significantly higher probability of obesity (two-proportion z-test: *P* < 0.05). The median age of cats for the 32 most common precise-level diagnoses varied from 0.82 years for adverse reaction to vaccination to 14.41 years for chronic kidney failure (Table 2).

**Table 2:** Prevalence of the most common disorders at precise-level diagnostic precision in Ragdoll cats (*n* = 2,025) under primary veterinary care at practices participating in the VetCompass programme in the UK from January 1st to December 31 st, 2019. P-values < 0.05 shown in bold. *Comparing the prevalence in males and females with two-proportion z-test

Precise-level disorder term	No.	Prevalence % (95% confidence interval)	Female prevalence %	Male prevalence %	*P*- value*	Median age [years] (range)
Periodontal disease	179	8.84 (7.60–10.08.60.08)	9.01	8.73	0.883	6.99 (0.84–18.35)
Diarrhoea	144	7.11 (5.99–8.23)	6.04	8.08	0.092	1.37 (0.26–15.84)
Obesity	140	6.91 (5.81–8.02)	5.09	8.54	**0.003**	3.53 (0.49–13.08)
Overgrown nail(s)	115	5.68 (4.67–6.69)	5.30	6.04	0.540	2.54 (0.23–19.51)
Dental disease	113	5.58 (4.58–6.58)	4.98	6.13	0.308	6.65 (0.54–19.00)
Flea infestation	91	4.49 (3.59–5.40)	5.41	3.62	0.067	1.58 (0.24–15.84)
Vomiting	82	4.05 (3.19–4.91)	3.82	4.27	0.687	3.74 (0.31–19.33)
Hair coat disorder	81	4.00 (3.15–4.85)	3.92	4.09	0.943	5.40 (0.73–18.31)
Conjunctivitis	51	2.52 (1.84–3.20)	2.65	2.41	0.844	1.32 (0.26–15.45)
Aggression	44	2.17 (1.54–2.81)	1.59	2.69	0.124	4.90 (0.80–13.74)
Anorexia	38	1.88 (1.29–2.47)	1.27	2.41	0.085	4.45 (0.48–19.07)
Thin	32	1.58 (1.04–2.12)	1.91	1.21	0.272	3.84 (0.31–18.31)
Ocular discharge	29	1.43 (0.91–1.95)	1.27	1.58	0.697	1.51 (0.26–14.79)
Weight loss	29	1.43 (0.91–1.95)	1.17	1.67	0.445	9.76 (2.24–18.41)
Postoperative wound complication	28	1.38 (0.87–1.89)	2.23	0.65	**0.005**	1.25 (0.24–5.49)
Aural discharge	26	1.28 (0.79–1.77)	1.91	0.74	**0.034**	1.18 (0.35–11.68)
Cat flu	24	1.19 (0.71–1.66)	1.38	1.02	0.594	1.04 (0.43–18.31)
Heart murmur	24	1.19 (0.71–1.66)	1.06	1.30	0.772	8.43 (0.38–18.35)
Disorder not diagnosed	24	1.19 (0.71–1.66)	1.38	1.02	0.594	9.72 (0.54–19.00)
Wound	24	1.19 (0.71–1.66)	0.85	1.49	0.266	2.71 (0.34–11.73)
Chronic kidney failure	23	1.14 (0.67–1.60)	1.17	1.11	> 0.999	14.41 (8.29–19.33)
Inappropriate urination	20	0.99 (0.56–1.42)	0.95	1.02	> 0.999	4.12 (0.31–15.45)
Sneezing	20	0.99 (0.56–1.42)	1.06	0.93	0.941	0.93 (0.36–12.50)
Hairball/furball	19	0.94 (0.52–1.36)	0.74	1.11	0.527	4.90 (0.93–13.59)
Abscess	18	0.89 (0.48–1.30)	0.74	1.02	0.668	3.51 (0.71–11.58)
Ear mites	17	0.84 (0.44–1.24)	0.74	0.93	0.831	1.71 (0.30–5.44)
Cystitis	16	0.79 (0.40–1.18)	1.17	0.46	0.127	7.95 (0.48–19.51)
Coughing	15	0.74 (0.37–1.11)	0.42	1.02	0.194	2.72 (0.73–18.49)
Upper respiratory tract infection	15	0.74 (0.37–1.11)	0.74	0.74	> 0.999	1.45 (0.30–13.54)
Adverse reaction to vaccination	14	0.69 (0.33–1.05)	0.74	0.65	> 0.999	0.82 (0.31–13.59)
Lameness	14	0.69 (0.33–1.05)	0.53	0.84	0.578	2.05 (0.26–15.51)
Overgrooming	13	0.64 (0.29–0.99)	0.64	0.65	> 0.999	6.76 (1.54–11.54)

There were 56 group-level disorders reported during 2019, of which the most common were dental (*n* = 294, prevalence 14.52%, 95% CI: 12.98–16.05), enteropathy (*n* = 273, prevalence 13.48%, 95% CI: 11.99–14.97), skin (*n* = 157, prevalence 7.75%, 95% CI: 6.59–8.92), obesity (*n* = 140, prevalence 6.91%, 95% CI: 5.81–8.02), and claw/nail (*n* = 125, prevalence 6.17%, 95% CI: 5.12–7.22) (Table 3). Among the 20 most common group-level disorders, females had higher probability of complications associated with clinical care, while males had higher probability of enteropathy, obesity, and traumatic injury (*P* < 0.05, two-proportion z-test). The median age of cats with the most common group-level disorders ranged from 1.37 years for complications associated with clinical care to 14.63 years for kidney disorder (Table 3).

**Table 3:** Prevalence of the most common disorders at group-level diagnostic precision in Ragdoll cats (*n* = 2,025) under primary veterinary care at practices participating in the VetCompass programme in the UK from January 1st to December 31 st, 2019. P-values < 0.05 shown in bold. *Shown as % (95% confidence interval) **Comparing the prevalence in males and females with two-proportion z-test

Diagnosis	No.	Prevalence (%)*	Female prevalence (%)*	Male prevalence (%)*	*P*- value	Median age (years) of cats with the condition
Dental	294	14.52 (12.98–16.05)	13.68	15.32	0.327	6.62 (0.54–19.00)
Enteropathy	273	13.48 (11.99–14.97)	11.77	15.04	**0.038**	2.33 (0.26–19.33)
Skin	157	7.75 (6.59–8.92)	7.74	7.80	> 0.999	5.23 (0.58–18.49)
Obesity	140	6.91 (5.81–8.02)	5.09	8.54	**0.003**	3.53 (0.49–13.08)
Claw/nail	125	6.17 (5.12–7.22)	5.83	6.50	0.597	2.86 (0.23–19.51)
Parasite infestation	119	5.88 (4.85–6.90)	6.57	5.20	0.223	1.62 (0.24–15.84)
Ophthalmological	117	5.78 (4.76–6.79)	5.83	5.66	0.947	1.58 (0.26–17.54)
Respiratory tract	86	4.25 (3.37–5.13)	3.82	4.64	0.420	2.54 (0.26–19.51)
Behavioural	79	3.90 (3.06–4.74)	3.39	4.36	0.314	4.59 (0.31–18.31)
Thin	61	3.01 (2.27–3.76)	3.08	2.88	0.898	7.61 (0.31–18.41)
Urinary system	53	2.62 (1.92–3.31)	3.29	2.04	0.108	6.72 (0.48–19.51)
Appetite	49	2.42 (1.75–3.09)	1.70	3.06	0.065	4.72 (0.48–19.07)
Traumatic injury	46	2.27 (1.62–2.92)	1.06	3.34	**0.001**	2.31 (0.34–14.77)
Heart	41	2.02 (1.41–2.64)	1.70	2.32	0.404	9.87 (0.38–19.51)
Ear	40	1.98 (1.37–2.58)	2.44	1.58	0.221	1.80 (0.35–13.68)
Complication associated with clinical care	39	1.93 (1.33–2.52)	2.76	1.21	**0.018**	1.37 (0.24–5.49)
Kidney	39	1.93 (1.33–2.52)	1.91	1.95	> 0.999	14.63 (2.51–19.33)
Mass/lump	38	1.88 (1.29–2.47)	2.12	1.67	0.563	11.25 (0.61–20.90)
Musculoskeletal	37	1.83 (1.24–2.41)	1.38	2.23	0.210	8.38 (0.26–18.31)
Lethargy	33	1.63 (1.08–2.18)	1.70	1.49	0.841	3.64 (0.67–15.33)

**Fig. 3:**
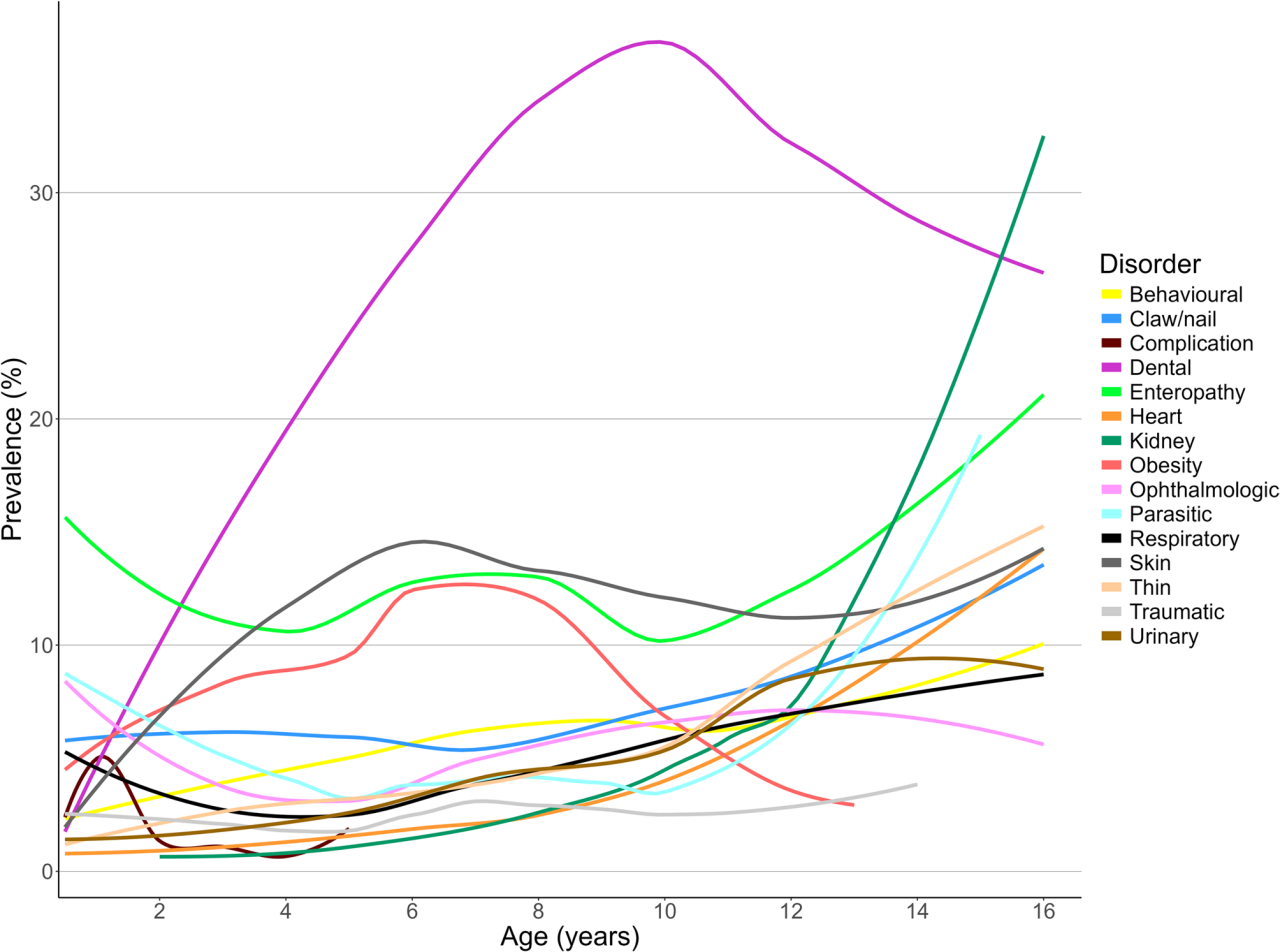
Annual prevalence of the combined list of 15 disorders from the 10 most common group-level disorders within each of three age bands (under 2 years *n* = 840, 2–6 years *n =* 744, 7 years or older *n* = 430) in Ragdoll cats under primary veterinary care at UK practices participating in the VetCompass Programme from January 1st to December 31 st, 2019

The prevalence of the 20 most common group-level disorders within each of the three age bands: < 2 years (*n* = 840), 2–6 years (*n* = 744), and ≥ 7 years (*n* = 430) is presented in Supplementary Table 1. The prevalence of all disorders except claw/nail disorders, ear disorders, lethargy, respiratory tract disorders, and traumatic injury varied significantly between the age groups (chi-square test, *P* < 0.05). Figure 3 shows the annual prevalence of each disorder in the full merged list from the 10 most common group-level disorders from each age band.

### Cause-specific mortality

In total, 133/2,025 (6.57%) of the Ragdolls died during the study period. The median age at death overall was 12.85 years (IQR 5.71–16.11, range 0.08–21.51). Life expectancy did not differ statistically between females and males (median age at death 13.72 years, IQR 6.77–16.42, range 0.18–21.51, *n* = 58, and 11.41 years, IQR 5.34–16.08, range 0.08–19.11, *n* = 75, respectively, Mann-Whitney U test, *P* = 264). Of 107 (80.45%) deaths with a recorded method of death, 98 (91.59%) involved euthanasia and 9 (8.41%) died unassisted.

Among 85 (63.91%) deaths with a cause reported, the most common causes of death at group level precision were kidney disorder (*n* = 18, 21.18%, 95% CI: 12.49–29.86) and poor quality of life (*n* = 11, 12.94%, 95% CI: 5.81–20.08) (Table 4).

**Table 4:** Cause-specific mortality in 85 Ragdoll cats with a recorded cause of death under primary veterinary care at UK practices participating in the VetCompass programme from January 1st to December 31 st, 2019. * Group-level disorders with ≥ 2 individuals **CI confidence interval

Group-level disorder*	No.	% (95% CI)**
Kidney	18	21.18 (12.49–29.86)
Poor quality of life	11	12.94 (5.81–20.08)
Mass/lump	5	5.88 (2.54–13.04)
Neoplasia	5	5.88 (2.54–13.04)
Respiratory tract	5	5.88 (2.54–13.04)
Traumatic injury	5	5.88 (2.54–13.04)
Brain	4	4.71 (1.85–11.48)
Collapsed	4	4.71 (1.85–11.48)
Enteropathy	4	4.71 (1.85–11.48)
Spinal cord	3	3.53 (1.21–9.87)
Appetite	2	2.35 (0.65–8.18)
Behaviour	2	2.35 (0.65–8.18)
Incontinence	2	2.35 (0.65–8.18)
Viral/infectious	2	2.35 (0.65–8.18)
Other	13	15.29 (7.64–22.95)

## Discussion

The current paper provides a comprehensive overview of the demography, disorders profile, life expectancy and cause-specific mortality of Ragdoll cats in the UK. Cats are increasingly recognised as a key companion animal species, with an estimated 10.6–10.8 million cats kept as pets in the UK, and 24–25% of UK adults owning at least one cat in 2024 [5, 28]. Historically, the majority of pet cats owned in the UK were crossbred (moggies) [29] but ownership of purebred cats (i.e. cats with a recognisable breed) has been reported as rising rapidly in recent years, from 35% of all cats in 2021 to 45% of all cats in 2024 [5]. The current results report that Ragdolls are one of the most common of the UK-owned cat breeds, making up 1.70% of all UK cats. Even more relevant, the results show that Ragdolls are rapidly rising in popularity, having risen almost five-fold in popularity from 0.70% of all cats born in 2005 to 3.72% in 2019. However, concerns have been raised about the lack of robust evidence on breed health available to prospective cat owners when deciding what type of cat to acquire, with 37% of owners who acquired a cat in 2024 reporting that the background information and the actual purchase relied on information sourced from social media [5]. Therefore, the development and publication of good evidence can help better inform the general public about the relative health and disorders of individual cat breeds and hopefully direct public opinion and actions towards prioritising the welfare of cats when making cat acquisition decisions [30].

Ragdolls were reported with published evidence on predispositions to 13 disorders in a disorder predisposition textbook. These predispositions included undesirable behaviour, aortic thromboembolism, hypertrophic cardiomyopathy, feline gastrointestinal eosinophilic sclerosing fibroplasia, cryptococcus, feline infectious peritonitis (FIP), cutaneous mast cell tumour, blood group incompatibility, dystocia, pyometra and also calcium oxalate, struvite and urate urolithiasis [9]. However, it is notable that few of these predispositions featured among the current results as being commonly diagnosed disorders of Ragdolls. This predisposition-vs-prevalence contrast highlights the importance of considering disorder each of prevalence and disorder predisposition as distinct concepts whereby prevalence offers information on absolute risk while predisposition offers information on relative risk [31].

Periodontal disease was the most common disorder recorded in Ragdoll cats in the current study, diagnosed in 8.84% of the cats during 2019. Periodontal disease also ranked as the most common disorder of cats overall in the UK in a previous study that used a similar method to the current study, although that study reported a higher prevalence of 15.2% in cats overall [3]. In line with this high frequency but apparent relative protection to periodontal disease for Ragdoll cats, another previous study using a similar method to the current study that specifically explored periodontal in cats reported a 9.4% prevalence for Ragdolls and that Ragdolls has 0.57 times the odds of periodontal disease compared to crossbred cats [32]. Periodontal disease in cats has been linked with increased risk of developing chronic kidney disease and azotaemia [33, 34] as well as also affecting the animal’s quality of life and interactions with the owner [35, 36]. Paradoxically, the high frequency and welfare impact from periodontal disease suggests that the condition should still be considered a welfare priority disorder in Ragdoll cats even though the breed appears to be protected to the condition.

Diarrhoea, with a prevalence of 7.11%, was the second most common disorder recorded in Ragdolls in the current study, with vomiting recorded in 4.05% of the Ragdolls as the 7th most common disorder. These values are higher than previously recorded for cats overall in the UK where vomiting (8th most common disorder) was recorded in 3.2% and diarrhoea (10th most common disorder) was recorded in 2.9% of cats [3]. These findings suggest the Ragdoll may be predisposed to enteropathy but do not confirm an aetiopathological pathway for such a predisposition. There is some evidence for predisposition to feline infectious peritonitis (FIP) caused by feline coronavirus (FCoV) infection in Ragdoll cats, which may contribute to a higher rate of diarrhoea and vomiting in the breed [37]. In a study of Japanese cats, 88.10% of Ragdoll cats were seropositive for FCoV compared to 31.2% of crossbred cats [38]. When symptomatic, FCoV often causes mild self-resolving enteropathy within clinical signs including diarrhoea [39]. Otherwise, FCoV is of little clinical significance unless the virus mutates to cause feline infectious peritonitis (FIP) which is a highly fatal and currently incurable systemic disease [40]. Higher prevalence of FCoV in the Ragdoll population could partially explain the increase in diarrhoea and enteropathies.

Haircoat disorder such as matting was recorded for 4.00% of the Ragdoll cats in the current study, making this the 8th most common disorder in the breed. This result suggests that the Ragdoll is predisposed to haircoat disorders which were recorded in just 2.6% of all cats in the UK and was the 10th most common disorder in a study using similar methods to the current study [3]. The Ragdoll breed is reported to have been invented by crossing between Persian and Angora cat types that both show a long-haired phenotype [2]. Extreme conformation has been defined as ‘a physical appearance that has been so significantly altered by humankind away from the ancestral natural appearance that affected animals commonly suffer from poor health and welfare, with negative impacts on their quality and quantity of life’ [8]. Haircoat disorder, with a prevalence of 12.7% that was over 3 times higher than for Ragdolls, was reported as the most common disorder of Persian cats in the UK, in a study using a similar method to the current study [41]. This high prevalence in Persian cats suggests that the long-haired phenotype should be considered an extreme conformation in cats and therefore should no longer be selected for if the goal is to select for good innate health [42]. Mutations in the Fibroblast Growth Factor 5 (FGF5) gene have been elucidated to explain the genetic inheritance for the long-haired phenotype in cats in an autosomal recessive manner, with this genetic understanding offering a pathway to select against this extreme conformation [43]. Although the modern Ragdoll is considered as a semi-longhaired breed, there are still warnings from cat organisations that the breed typically has a coat that knots easily, especially under the front and hind legs, if the cat is not regularly groomed, with this predisposition to haircoat disorder supported by the current results [2].

The current results based on 2,025 Ragdolls highlight that age is a major risk factor for many common disorders. Figure 3 presents annual prevalence for 15 disorder groups that featured among the 10 most common group-level disorders within three age bands: < 2 years, 2–6 years and ≥ 7 years. The prevalence varied significantly between these age bands for 12 of the 15 disorders, with only claw/nail disorders, respiratory tract disorders and traumatic injury not showing a statistically significant difference. These findings align with a previous larger study of 18,249 cats of all breeds in the UK that used a similar study design to report an age effect for all 30 grouped-level disorders assessed [3]. It is possible that a failure to detect an age effect for the three disorders in the current study may reflect underpowering due to a smaller sample size or alternatively that the age effects for disorders in Ragdolls genuinely differs to the wider cat population. The age-based prevalence pattern for dental disorder is notable in the current results for showing a very steep rise to a very high prevalence of over 35% around 9 years and then reducing as age advances beyond this point. A previous study of periodontal disease across all types of cats under primary veterinary care in the UK using the same methodology showed a similar steep rise odds up to nine years of age [32]. However, in that study, a high odds of periodontal disease was maintained from nine years onwards, suggesting the pattern of dental disease in older Ragdolls may differ to that of cats in general in the UK but the reasons for any differences here are unclear.

The 12.85 years median age at death for Ragdolls in the current study is more than one year shorter than the 14.0 years previously recorded for cats overall in the UK in a study that used a similar method [12]. However, that study of 4,009 cat deaths that included 91.7% crossbred cats also identified a substantial survival benefit for crossbred cats (median age at death 14.0 years) over purebred cats (median age at death 12.5 years). Therefore, the current results suggest that the Ragdoll life expectancy is largely typical for purebred cats overall and that much of the reduced life expectancy in Ragdolls relative to cats overall is likely a consequence of being purebred rather than specifically related to being a Ragdoll compared to any other individual purebred. Similar reduced longevities have been reported for purebred dogs compared to crossbred dogs, with the underlying reasons for this reduced life expectancy linked to higher levels of inbreeding and extreme conformations in the purebred animals overall [44, 45].

The current study has some limitations. The study reports on absolute disorder risk within Ragdoll cats but future work that also extracted disorder data on the remaining non-Ragdoll cats in the underlying population could support formal reporting of breed predispositions as have been published for some dog breeds [46–48]. A more complete welfare impact assessment than is given in the current study would require extraction of disorder duration and severity data in addition to the current prevalence data [49]. The current study was unable to distinguish between Ragdoll cats that were registered with pedigrees and those that were not. The current data relied on accurate record-keeping and high clinical acumen of the primary care veterinary teams. The consistency of recording of coat colours in the current clinical records was not considered high enough to support reporting and analysis of coat colour in the current paper. The data included in the final analysis was also determined by decision-making by the research terms on data cleaning criteria, such as maximal bodyweights and ages to allow for the analysis [50]. The retrospective nature of the study design (whereby the study was designed and executed after the study data had been recorded by the participating veterinary clinics) did not allow for the collection and validation of additional data fields such as body condition score that might have been possible with prospective data collection [51].

## Conclusions

The current study reports the Ragdoll as a relatively common purebred cat breed in the UK and with steeply rising levels of ownership that support the value of increased emphasis on evidence generation on the demography, disorder profiles and cause-specific mortality of the breed. The most common disorder was periodontal disease but the breed appears predisposed to haircoat disorder and enteropathy. While the median age at death of 12.85 years is shorter than for cats overall, this value was in line with the relatively shorter life expectancy for purebred cats overall in the UK. The Ragdoll cat breed appears to be largely typical in terms of disorders and cause-specific mortality for purebred cats overall in the UK.

## Supplementary Information

Supplementary material 1.

## Data Availability

The dataset supporting the conclusions of this article are openly available on Figshare 10.6084/m9.figshare.28103699.

## Abbreviations

CI: Confidence interval
EHR: Electronic health record
FCoV: Feline coronavirus
FGF5: Fibroblast growth factor 5
FIP: Feline infectious peritonitis
GCCF: Governing Council of the Cat Fancy
IQR: Interquartile range
PAAG: The Pet Advertising Advisory Group
