# Supplementary material for: Demography, common disorders, cause-specific mortality and life expectancy of Ragdoll cats under primary veterinary care during 2019 in the UK
Source: Companion Anim Health Genet. 2025 Nov 5;12:8. doi: 10.1186/s40575-025-00148-9 (PMC12587670; doi:10.1186/s40575-025-00148-9)
Supplement: Supplementary file 1 — Supplementary material 1. [file 40575_2025_148_MOESM1_ESM.docx]

Supplementary Table A. Frequency, prevalence and rank order of the 20 most common disorders at group-level diagnostic precision in each of three age bands: < 2 years (n = 840), 2-6 years (n = 744), and ≥ 7 years (n = 430) in Ragdolls (n = 2,025) under primary veterinary care at practices participating in the VetCompass Programme in the UK from January 1^st^ to December 31^st^, 2019. **CI* confidence interval **Comparison of prevalence values between each age band, using chi-square test. Significant *P-* values are in bold

| Diagnosis | No. < 2 years | No. 2-6 years | No ≥ 7 years | Prevalence % (95% CI*) < 2 years | Prevalence % (95% CI*) 2-6 years | Prevalence % (95% CI*) ≥ 7 years | Rank < 2 years | Rank 2-6 years | Rank ≥ 7 years | *P-* value** |
| --- | --- | --- | --- | --- | --- | --- | --- | --- | --- | --- |
| Appetite | 14 | 13 | 21 | 1.67 (0.80-2.53) | 1.75 (0.81-2.69) | 4.88 (2.85-6.92) | 14 | 15 | 14 | **< 0.001** |
| Behavioural | 20 | 32 | 27 | 2.38 (1.35-3.41) | 4.30 (2.84-5.76) | 6.28 (3.99-8.57) | 11 | 7 | 8 | **0.003** |
| Claw/nail | 47 | 48 | 30 | 5.60 (4.04-7.15) | 6.45 (4.69-8.22) | 6.98 (4.57-9.38) | 4 | 5 | 7 | 0.590 |
| Complication associated with clinical care | 31 | 8 | 0 | 3.69 (2.42-4.97) | 1.08 (0.55-2.11) | 0 | 7 | 19 | - | **< 0.001** |
| Dental | 24 | 131 | 139 | 2.86 (1.73-3.98) | 17.61 (14.87-20.34) | 32.33 (27.90-36.75) | 9 | 1 | 1 | **< 0.001** |
| Ear | 20 | 15 | 5 | 2.38 (1.35-3.41) | 2.02 (1.01-3.03) | 1.16 (0.50-2.69) | 12 | 12 | 27 | 0.337 |
| Enteropathy | 131 | 78 | 63 | 15.60 (13.14-18.05) | 10.48 (8.28-12.69) | 14.65 (11.31-17.99) | 1 | 2 | 2 | **0.009** |
| Heart | 6 | 9 | 25 | 0.71 (0.33-1.55) | 1.21 (0.64-2.28) | 5.81 (3.60-8.03) | 23 | 17 | 9 | **< 0.001** |
| Kidney | 0 | 3 | 36 | 0 | 0.40 (0.14-1.18) | 8.37 (5.75-10.99) | - | 27 | 4 | **< 0.001** |
| Lethargy | 12 | 8 | 12 | 1.43 (0.63-2.23) | 1.08 (0.55-2.11) | 2.79 (1.23-4.35) | 16 | 20 | 17 | 0.068 |
| Mass/lump | 4 | 9 | 24 | 0.48 (0.19-1.22) | 1.21 (0.64-2.28) | 5.58 (3.41-7.75) | 26 | 18 | 11 | **< 0.001** |
| Musculoskeletal | 11 | 7 | 19 | 1.31 (0.54-2.08) | 0.94 (0.46-1.93) | 4.42 (2.48-6.36) | 18 | 21 | 15 | **< 0.001** |
| Obesity | 43 | 65 | 32 | 5.12 (3.63-6.61) | 8.74 (6.71-10.77) | 7.44 (4.96-9.92) | 5 | 4 | 6 | **0.017** |
| Ophthalmological | 68 | 24 | 24 | 8.10 (6.25-9.94) | 3.23 (1.96-4.50) | 5.58 (3.41-7.75) | 2 | 8 | 12 | **< 0.001** |
| Parasite infestation | 67 | 39 | 12 | 7.98 (6.14-9.81) | 5.24 (3.64-6.84) | 2.79 (1.23-4.35) | 3 | 6 | 18 | **< 0.001** |
| Respiratory tract | 39 | 23 | 24 | 4.64 (3.22-6.07) | 3.09 (1.85-4.34) | 5.58 (3.41-7.75) | 6 | 9 | 13 | 0.100 |
| Skin | 27 | 70 | 58 | 3.21 (2.02-4.41) | 9.41 (7.31-11.51) | 13.49 (10.26-16.72) | 8 | 3 | 3 | **< 0.001** |
| Thin | 7 | 20 | 33 | 0.83 (0.40-1.71) | 2.69 (1.53-3.85) | 7.67 (5.16-10.19) | 21 | 10 | 5 | **< 0.001** |
| Traumatic injury | 22 | 15 | 9 | 2.62 (1.54-3.70) | 2.02 (1.01-3.03) | 2.09 (1.10-3.93) | 10 | 13 | 21 | 0.694 |
| Urinary system | 12 | 16 | 25 | 1.43 (0.63-2.23) | 2.15 (1.11-3.19) | 5.81 (3.60-8.03) | 17 | 11 | 10 | **< 0.001** |
